# Quality of Protection Evaluation of Security Mechanisms

**DOI:** 10.1155/2014/725279

**Published:** 2014-07-17

**Authors:** Bogdan Ksiezopolski, Tomasz Zurek, Michail Mokkas

**Affiliations:** ^1^Institute of Computer Science, Maria Curie-Sklodowska University, Plac Marii Curie-Sklodowskiej 5, 20-031 Lublin, Poland; ^2^Polish-Japanese Institute of Information Technology, Koszykowa 86, 02-008 Warsaw, Poland

## Abstract

Recent research indicates that during the design of teleinformatic system the tradeoff between the systems performance and the system protection should be made. The traditional approach assumes that the best way is to apply the strongest possible security measures. Unfortunately, the overestimation of security measures can lead to the unreasonable increase of system load. This is especially important in multimedia systems where the performance has critical character. In many cases determination of the required level of protection and adjustment of some security measures to these requirements increase system efficiency. Such an approach is achieved by means of the quality of protection models where the security measures are evaluated according to their influence on the system security. In the paper, we propose a model for QoP evaluation of security mechanisms. Owing to this model, one can quantify the influence of particular security mechanisms on ensuring security attributes. The methodology of our model preparation is described and based on it the case study analysis is presented. We support our method by the tool where the models can be defined and QoP evaluation can be performed. Finally, we have modelled TLS cryptographic protocol and presented the QoP security mechanisms evaluation for the selected versions of this protocol.

## 1. Introduction

Balancing security against performance in IT systems is one of the important issues to be solved. The traditional approach assumes that the best way is to apply the strongest possible security measures, which makes the system as secure as possible. Unfortunately, such reasoning can lead to the overestimation of security measures which causes an unreasonable increase in the system load [1, 2]. This problem is especially important in multimedia systems. The example from [2] may relate to the audio/video streaming as the real-time service. This teleconference can be protected by the VPN data transmission which will be accomplished by the TLS tunneling. After the service requirements analyses one can assign different versions of the protocol depending on the level of protection. The selection is presented in Table 1. In the first version one chooses the RC2-CBC algorithm and MD5 hash function. In the second version one selects the strongest symmetric algorithm (DES-CBC) and hash function with the longest digest (SHA1). In the third version one selects the symmetric algorithm with the key 3DES-CBC longer than the one selected in the second version.

In [2] authors checked how the security mechanism influences the efficiency of the peers during the video teleconference. The speed of transmitting data in the video conference which was secured by the VPN connection was checked. The results are presented in Table 2. The required quality of the video conference can be guaranteed only if we can transmit 240 KB/s and simultaneously receive the same amount of data. The transfer of the required level (480 KB/s) is guaranteed by the first version of the protocol (low). For the second version (medium) it is equal to the required bit rate. The third version of the protocol, which accomplished the VPN connection on the high level, cannot make the video conference of the required quality. The presented results show that overestimation of security mechanisms during data transmission leads to the decreasing efficiency of the devices from which the transmission is accomplished.

The system performance is also important in the systems with limited resources, such as wireless system networks or mobile devices. Another example where such an analysis should be performed is cloud architecture. The latest research indicates that the three main barriers for using cloud computing are security, performance, and availability [3]. Unfortunately, when the strongest security mechanisms are used, system performance decreases and system availability is further influenced. The solution is using the QoP models. The latest results show [2, 4–6] that in many cases the better way is determination of the required level of protection and adjustment of some security measures to these requirements. Such an approach is achieved by means of the quality of protection models where the security measures are evaluated according to their influence on system security.

One of the most challenging issues in all QoP models is performing quality of protection evaluation of security mechanisms for the different versions of the cryptographic protocol (security policies). All of the approaches [4, 7–9] introduce different formulae which estimate the influence of security mechanisms for QoP, but they also have one significant limitation. These models can evaluate only these versions which were previously directly defined and described in an evaluation system. As directly defined scenarios we understand previously predefined models of configurations of security mechanisms which secure the IT processes. Unfortunately, defining all possible scenarios for all IT processes is very complex and in many cases is not feasible. The result of a nondefined scenario can be the situation in which security mechanisms of a specific IT process would not be evaluated; for example, adding a new security mechanism to an existing system may entail its lack of adjustment to any existing scenario and, consequently, the failure of its evaluation. The QoP evaluation of security mechanisms can be performed as part of risk analysis process or can be part of the decision support system, which in an adaptable way define appropriate configuration of security mechanisms. As a result of the lack of QoP evaluation of security mechanisms, the decision support system would not perform action adequate to the situation. This limitation is especially important in real-time systems [2, 10].

In the presented paper, we introduce a model of QoP evaluation of security mechanisms. Our main contribution is that the QoP evaluation of security mechanisms can be performed for not directly defined configurations of security mechanisms. The additional contribution of the paper is implementing the security mechanisms evaluation tool (SMETool) which supports the presented method. This tool can be used either by researchers or by security engineers. The SMETool can be downloaded from the web page of the Quality of Protection Modelling Language Project [11].

The paper is organized as follows. In the second section the related work about QoP models is presented. In the third section the formal model definition is presented. In the fourth section the methodology of QoP evaluation of security mechanisms is described. The fifth section deals with the case study of QoP evaluation of security mechanisms, where the TLS handshake protocol is presented. Finally, section six includes the conclusions.

## 2. Related Work

In the literature the security adaptable models are introduced as the quality of protection (QoP) models [4, 7–9, 12–17]. These models were created for different purposes and have different features and limitations. The related research in this area is presented below.

Lindskog attempt to extend the security layers in a few quality of service (QoS) architectures [14]. Unfortunately, the descriptions of the methods are limited to the confidentiality of data and based on different configurations of the cryptographic modules. Ong et al. in [15] present the QoP mechanisms, which define security levels depending on security parameters. These parameters are as follows: key length, block length, and contents of an encrypted block of data. Schneck and Schwan [16] propose an adaptable protocol concentrating on the authentication. By means of this protocol, one can change the version of the authentication protocol which finally changes the parameters of the asymmetric and symmetric ciphers. Sun and Kumar [17] create the QoP models based on the vulnerability analysis which is represented by the attack trees. The leaves of the trees are described by means of the special metrics of security. These metrics are used for describing individual characteristics of the attack. In [4] Ksiezopolski and Kotulski introduce mechanisms for adaptable security which can be used for all security services. In this model the quality of protection depends on the risk level of the analysed processes. Luo et al. [8] provide the quality of protection analysis for the IP multimedia systems (IMS). This approach presents the IMS performance evaluation using Queuing Networks and Stochastic Petri Nets. LeMay et al. [13] create the adversary-driven, state-based system security evaluation, the method which quantitatively evaluates the strength of systems security. In [9] Petriu et al. present the performance analysis of security aspects in UML models. This approach takes as an input of a UML model of the system designed by the UMLsec extension [18] of the UML modelling language. This UML model is annotated with the standard UML profile for schedulability, performance, and time and then analysed for performance. In [12] Ksiezopolski introduce the quality of protection modelling language (QoP-ML) which provides the modelling language for making abstraction of cryptographic protocols that put emphasis on the details concerning quality of protection. The intended use of QoP-ML is to represent the series of steps which are described as a cryptographic protocol. The QoP-ML introduced the multilevel [19, 20] protocol analysis that extends the possibility of describing the state of the cryptographic protocol. In [7] the authors present the impact of used security mechanisms in wireless local area networks for its quality of service. In this approach the QoP model is introduced which quantifies the benefits of security policies and demonstrates the relationship between QoS and QoP.

## 3. Model

Main aim of the model is to create a tool which helps to evaluate the quality of security protection of a given IT system. We are going to achieve it by evaluation of a set of security attributes of a given system, where as security attributes we understand various aspects of protection of a given system. At the beginning of the evaluation process we have a system which can be described by a set of facts. These facts represent all the elements of a given system. On the grounds of a set of facts we may, based on knowledge base, knowledge representation mechanism, and expert system-like forward chaining mechanism, infer a more general description of a given system and perform evaluation of the quality of protection of the analyzed system. The model is a semiformal tool which should allow to represent features of the analyzed system, as well as knowledge required to perform evaluation of the quality of protection of the system. The model also assumes the utilisation of the inference mechanisms which are a slightly modified version of expert systems like forward chaining mechanisms.

The goals of the presented model are as follows.

1. The QoP evaluation of security mechanisms can be performed for not directly defined scenarios.
2. Making quality of protection evaluation of all security mechanisms possible.
3. Analysis refers to all security attributes.
4. The model can be used for any QoP models.

### 3.1. Facts and Rules

We assume that the system behaviour modelled in one of the QoP models is represented by means of a set of propositions which we call facts:

(1) $$\begin{array}{c} F=\left\{f_{1},f_{2},f_{3},\ldots ,f_{n}\right\}, \end{array}$$

where *f*_1_,…, *f*_*n*_ are the facts describing a system.

By means of the facts one can define any security mechanisms. We assume a set of operators OP = {¬, ~, ∨, ∧, ⇒, →}, where

- ¬ is a classical (strong) negation;
- ~ is a negation as failure;
- ∨ is a disjunction;
- ∧ is a conjunction;
- ⇒ is a defeasible implication;
- → is a strict implication.

**Definition 1 (literals).**

Facts (negated in a strict way or nonnegated) are literals. The set of all literals is *L* = {*l*_1_, *l*_2_,…, *l*_*m*_}.

For example if *F* = {*f*_1_, *f*_2_} is a set of facts then *L* = {*f*_1_, *f*_2_, ¬*f*_1_, ¬*f*_2_} is a set of literals.

**Definition 2 (case).**

A case is a model of an evaluated system represented by a set of literals *C* = {*l*_*a*_, *l*_*b*_,…, *l*_*s*_, *l*_*z*_,…}, which may be also expressed by a set of positive or negated facts: *C* = {*f*_*a*_, *f*_*b*_,…, ¬*f*_*s*_, ¬*f*_*z*_,…}.

**Definition 3 (security attribute).**

Security attribute is an attribute which describes system behaviour in the case of information security requirements.

For example, one can enumerate the following security attributes [4, 21, 22]: integrity, confidentiality, authentication, availability, or anonymity. The security attributes (SA) set consists of an unlimited but finite number of security attributes. Each of them has its own evaluation value expressed by a positive integer number. Evaluation value represents the estimation of its security attribute. A security attribute may have a positive or negative character, in the sense that bigger value of security attribute evaluation may mean better (for positive) or worse (for negative) evaluation.

**Definition 4 (rule).**

Rule is a formula in the following form:

(2) $$\begin{array}{c} \text{Conditions}⟶\text{Conclusion}, \end{array}$$

where

- Conditions is a list of rule conditions.A list of conditions is in the form *wl*_*a*_ *func* *wl*_*b*_ *func*   ⋯  *wl*_*d*_, where  * func*   is one of the operators from the set = {∨, ∧}, and {*wl*_*a*_, *wl*_*b*_,…, *wl*_*d*_} are the facts (nonnegated or negated by negation as failure). Only one kind of operators can be used in one rule.
- Conclusion is a rule conclusion in the form Conclusion = (*lx*∧*ly*∧⋯), where (*lx*∧*ly*∧⋯) ∈ *L*.

Conditions may be negated by negation as failure and conclusions may be negated by classical negation. In the antecedent part of the rule, it is forbidden to use classical negation. In the consequent part of the rule, it is forbidden to use negation as failure. The set of rules is denoted as *RF*.

Rules allow us to represent relations between various facts, because in real life systems the existence of a chosen feature causes the existence (or not) of some other features. They also allow us to express which facts are exclusive in the sense that the existence of one of them causes the nonexistence of others. It is important because it helps to preserve consistency of the model.

### 3.2. Evaluation Rules

The assessment of the security attributes of a given computer system is based on facts and evaluation rules.

**Definition 5 (evaluation rule).**

Evaluation rules are formulae in the following form:

(3) $$\begin{array}{c} \text{Conditions}⟹\mathrm{In}{\text{f}}^{V}\left(\text{sa}\right), \end{array}$$

where

- Conditions is a list of rule conditions in the form *wl*_*a*_ *func* *wl*_*b*_ *func*   ⋯  *wl*_*d*_, where  * func*   are the operators from the set = {∨, ∧} and {*wl*_*a*_, *wl*_*b*_,…, *wl*_*d*_} are facts (nonnegated or negated by negation as failure).
- In⁡f is a function changing value of evaluation of security attribute sa by adding value *V* (security influence) to the security attribute evaluation, when *V* is an integer number which represents the evaluation of a given security attribute.

Security attribute evaluation cannot be lower than 0. Special function In⁡f^0^(sa) means that the security attribute sa evaluation is reduced to 0. This function will reduce sa to 0 regardless of sa, the current value. When this operator is used, it means that this sa is not guaranteed. The *V* value of sa will be equal to 0 disregarding other evaluation rules which could increase *V* value. This mechanism will be described more precisely later.

It is important to notice that in fact evaluation rules are not rules in a traditional sense of this term. They are conditionals in which the satisfaction of their conditions causes change of value of evaluation attribute.

We denote the set of evaluation rules as a ER. The example of the evaluation rules set ER is

(4) $$\begin{array}{c} f_{1}\wedge f_{2}⟹\mathrm{In}{\text{f}}^{10}\left(\text{Confidentiality}\right),\\ f_{3}\wedge \sim f_{4}⟹\mathrm{In}{\text{f}}^{0}\left(\text{Integrity}\right). \end{array}$$

The fulfillment of the evaluation rule conditions causes an appropriate change of the security attribute evaluation value. For example, when we have the above rules and we have the case *P* = {*f*_1_, *f*_2_, *f*_3_}, we may conclude that the value of evaluation of security attribute Confidentiality should increase by 10 points and the value of evaluation of security attribute Integrity should decrease to 0 points.

The evaluation rule uses defeasible implication because such a rule may be defeated by another one.

**Definition 6 (strict satisfaction of rule conditions).**

Rule conditions are satisfied in a strict way if positive (nonnegated) conditions are true and negative conditions (facts negated with negation as failure) are false or it is impossible to conclude that they are true.

The example of the rule is

(5) $$\begin{array}{c} f_{1}\wedge f_{2}\wedge \sim f_{3}⟶f_{4}. \end{array}$$

From the above rule we may conclude that if *f*_1_ and *f*_2_ are true and *f*_3_ is false (it is not declared, it cannot be concluded from other rules, and it is declared that ¬*f*_3_ or there is a rule with the conclusion ¬*f*_3_ and its conditions are satisfied), then *f*_4_ is true.

#### 3.2.1. Orders between Facts

It is easy to notice that the evaluation of complex systems requires building of a large set of rules, which should allow for evaluation of any real life system. It is also possible that such a set of rules may be, due to many reasons, incomplete in the sense that there may be facts which are not used in any rule and evaluation rule.

Such a situation may lead the evaluation process to misleading consequences which come from the lack of evaluation of potentially important facts. We can also notice that such new facts unpredicted in the rule base may be in a way connected to the other ones which are already regulated. They may, for example, represent better satisfaction of a condition of a chosen rule. On the other hand it is sometimes much easier to declare that, for example, fact *f*_1_ means more than *f*_2_ and may satisfy the condition of a chosen rule in a better way.

Based on the above, we assume the possibility of the declaration of orders between facts. The partial order *f*_1_ > *f*_2_ denotes that *f*_1_ means more than *f*_2_ and if there is a rule in which *f*_2_ is one of the conditions and we know that *f*_1_ is satisfied, then we may conclude that *f*_2_ should also be satisfied (even if it is not literally true). Such an order represents better satisfaction of the rule condition. For example, if *f*_2_ means cipher with the default key length, *f*_1_ may denote cipher with the longer key length.

It is worth mentioning that these relations may not be the same for every security attribute. For example, a longer key length is better in the matter of confidentiality, but it is worse in the matter of efficiency. According to the above, we have to add to the order additional information about security attribute in which this reasoning concerns.

We also assume that the relation of order between facts is transitive:

(6) $$\begin{array}{c} \forall_{\left(X,Y,Z\right)}\left(\left(X>Y\right)\wedge \left(Y>Z\right)⟶\left(X>Z\right)\right). \end{array}$$

We introduce the structure OF: *OF* = 〈*F*, >_SA_〉, where >_SA_ is a relation of strict partial order which represents preferences between various facts from the set *F* in the context of security attribute SA (*f*_1_>_sa_ *f*_2_ denotes that *f*_1_ means more than *f*_2_ in the context of security attribute sa).

**Definition 7 (unstrict satisfaction of rule conditions).**

Condition *f*_*x*_ of a given rule is satisfied in an unstrict way when *f*_*x*_ is not true, but

- there is a fact *f*_*y*_;
- it is not known that ¬*f*_*x*_ (~¬*f*_*x*_);
- we know that *f*_*y*_>_SA_ *f*_*x*_;
- reasoning concerns evaluation of security attribute SA.

Such kind of satisfaction of the condition of the rule we call unstrict satisfaction of rule conditions. The root of unstrict satisfaction of the rule lies in the so called a'fortiori reasoning (reasoning from more to less), which is commonly used in legal domain. The example of formalisation of such a way of reasoning is presented in [23].

Looking more generally at the above, we may notice that falsehood of the condition is not sufficient to assume that it is not satisfied. In other words, we have to distinguish truthfulness of the condition from its satisfaction. In the model we assume that false condition may be, in the above mentioned cases, treated as satisfied one. We also assume that it is not possible to treat true condition as unsatisfied.

Another important thing which is connected to the above defined unstrict satisfaction of the rule conditions is a necessity of preservation of the consistency of the model of the system (as consistency we understand here the exclusion of the possibility of existing complementary facts, for example, *f*_1_, ¬*f*_1_). The second clause of the above definition (~¬*f*_*x*_) controlling condition may be satisfied in the unstrict way only if it is not known that it is false and it is impossible to derive that it is false. This clause determines that unstrict satisfaction may be defeated by strict declaration of another fact or by another rule.

**Definition 8 (satisfaction of the conditions of the rule).**

If there is a given case *C* described by a set of literals *C* = {*l*_*x*_, *l*_*y*_,…, *l*_*z*_} which satisfies in a strict or unstrict way conditions (Conditions) of a rule *rf* ∈ *RF*, then we denote it as *C*•Conditions.

### 3.3. Inference Rule

The above system has one important feature: there is a distinction between truthfulness of the condition and its satisfaction. In order to create an inference mechanism for our model, we have to modify the classic* Modus Ponens* rule.

**Definition 9 (inference rule).**

As an inference rule we understand the following rule:

(7) $$\begin{array}{c} \frac{\left(\text{Conditions}⟶\text{Conclusions}\right)\wedge f•\text{Conditions}}{\text{Conclusions}}, \end{array}$$

where → is a strict implication and *f*•Conditions mean that fact *f* satisfies (in a strict or unstrict way) conditions of a given rule.

Strict or unstrict satisfaction of the rule antecedents allows us to treat rule conclusion as true and, consequently, also satisfied.

### 3.4. Inference Mechanism

On the basis of the above defined inference rule, we have to define our inference mechanism.

**Definition 10 (fact based inference mechanism).**

As a fact based inference mechanism we understand forward chaining mechanism using the inference rule defined earlier. As *C*′ we denote a set of conclusions whose inference mechanism concludes from a case *C*, a set of rules *RF* and a set of orders *OF*. We may also denote it as *C*⊢*C*′. The union of sets *C* ∪ *C*′ we call a complete description of the case and denote as *P*.

### 3.5. Security Attributes

As described above, the security attributes' set SA consists of an unlimited but finite number of security attributes. Each of them has its own evaluation value expressed by a positive integer number.

**Definition 11 (set of security attributes pairs.).**

*S* is a set of pairs *O* = 〈sa, *o*〉, where sa ∈ SA is a security attribute and *o* is its evaluation value.

For example, we have three security attributes with their evaluation values:

(8) $$\begin{array}{c} \text{SA}=\text{confidentiality},\text{integrity},\text{authorisation};\\ S=\left\{\left(\text{confidentiality},10\right),\left(\text{integrity},20\right),\left(\text{author}.,30\right)\right\}. \end{array}$$

It is important to notice that security attributes may have positive or negative character, in the sense that a higher value of security attribute evaluation may mean better (for positive) or worse (for negative) evaluation.

### 3.6. Conflicts between Rules

Some specific conditions may cause conflicts between evaluation rules.

**Definition 12 (conflicting rules).**

There is a conflict between two or more evaluation rules if these rules cannot be executed together.

Such conflicts may appear when there are two rules whose antecedents are satisfied, who are in a way connected and the execution of both of them may cause improper influence on the security attribute evaluation.

The problem of conflicting and subsuming rules is the main reason for the utilisation of defeasible implication. In this work as defeasibility of the evaluation rules we understand the possibility of exclusion from the evaluation process of a chosen rule by another rule. If antecedents of two conflicting rules are satisfied, only one of them may be executed (but such a rule may be also defeated by another one).

To represent priorities between the evaluation rules we assume partial order between rules from a set *ER*. Such an order allows us to express that if *r*_1_ > *r*_2_ and *r*_1_, *r*_2_ ∈ *ER*, then rules *r*_1_ and *r*_2_ are in conflict and when the conditions of both of these rules are satisfied rule *r*_1_ should defeat rule *r*_2_.

#### 3.6.1. Reasoning about Orders between Conflicting Rules

The main problem of the above mentioned issue of conflicting rules lies in the mechanism of recognition of conflicting rules. Generally, such recognition should be based on commonsense reasons, but there is one phenomenon, which allows us to recognize conflict and to find order between conflicting rules. This situation concerns a case where there are two rules, one of which has a more general set of conditions than the other one. In other words, every case satisfying condition of the rule *r*_2_ also satisfies conditions of the rule *r*_1_. Such subsumption of the rules allows us to conclude that the rule *r*_2_ is a specific case of the rule *r*_1_, and in the case of satisfaction of the antecedents of both rules, the rule *r*_2_ should defeat the rule *r*_1_. More generally we may say that two rules are in conflict when the list of conditions of one of them subsumes that of the second one and the conclusions of both concern the modification of the same security attribute evaluation value.

**Definition 13 (subsuming rules).**

When we have the following two rules:

(9) $$\begin{array}{c} r_{x}:{\text{Condition}}_{x}⟹\mathrm{In}{\text{f}}^{V1}\left(\text{sa}\right),\\ r_{y}:{\text{Condition}}_{y}⟹\mathrm{In}{\text{f}}^{V2}\left(\text{sa}\right), \end{array}$$

where Condition_*x*_ and Condition_*y*_ are the lists of antecedents of these rules, both rules influence the same security attribute evaluation value sa (but they may have a different level of influence), and if for any case *P* represented by a set of literals:

(10) $$\begin{array}{c} \forall_{P\in L}\left(\left(P•\text{Conditio}{\text{n}}_{x}\right)⟶\left(P•\text{Conditio}{\text{n}}_{y}\right)\right), \end{array}$$

then we recognise the rules *r*_*x*_ and *r*_*y*_ as subsuming and conflicting ones and in the view of a more restrictive character of the rule *r*_*x*_ we may conclude that the rule *r*_*x*_ has priority over the rule *r*_*y*_, which we denote: *r*_*x*_ > *r*_*y*_ and while conditions of both rules are satisfied, the rule *r*_*x*_ should defeat the rule *r*_*y*_.

Rules with the function In⁡f^0^(sa) on the consequent part of the rule have the highest possible priority and they are in conflict with all the rules concerning the security attribute sa. Everytime when they satisfy conditions, they exclude all evaluation rules concerning the security attribute sa from reasoning.

Another aspect which is important and requires explanation is connected with the reason why a more specific rule defeats a more general one. Such a mechanism comes from the theory of law and is called* lex specialis derogat legi generali*. It is one of the tools which allow to find a solution in conflicting legal rules, saying that a specific act (provision) derogates from (prevails over) the general regulation. In modelling legal rules there are many problems connected with the difficulties with recognition which of the rules are more or less specific [24]. In the case of our model it is much simpler because the hierarchy of generality of antecedents of the rules is easy to establish based on a finite number of possible facts and their explicitness.

### 3.7. Evaluation Rules System

**Definition 14 (evaluation rules system).**

Evaluation rules system RO is a structure described by a set of evaluation rules ER and relation OR= 〈ER, >〉.

The relation OR represents partial order between rules from a set ER. This order maps preferences between conflicting rules. These preferences can come from strict declaration or from a previously defined mechanism of finding and resolving a problem of subsuming rules. If there is a relation of partial order between two rules, we treat them as conflicting ones. If these rules are not comparable, we treat them as conflict free. We also assume that the relation of order between rules is transitive:

(11) $$\begin{array}{c} \forall_{r_{x},r_{y},r_{z}}\left(\left(r_{x}>r_{y}\right)\wedge \left(r_{y}>r_{z}\right)⟶\left(r_{x}>r_{z}\right)\right). \end{array}$$

### 3.8. QoP Evaluation Process of Security Mechanisms

The process of QoP evaluation of security mechanisms is expressed by means of evaluation of security attributes. The values of the SA depend on the used security mechanisms which are represented in the model by facts *F*.

The evaluation process of security mechanisms may be described in terms of a sequence of steps as presented by Algorithm 1. The parameters and variables used in this algorithm are presented in Table 3.

### 3.9. Background of the Model

Looking more generally at our model, one can notice that it contains some elements taken from the formal models of legal reasoning. Legal reasoning has a very specific character, as it requires mechanisms of dealing with incomplete knowledge, mechanisms of resolving conflicts between legal rules and arguments, various ways of interpretation of rules, and so forth. Computer systems which aims to support human reasoning very often, have to face similar problems, which is the main reason for the utilisation of the* a'fortiori* rule or the* lex specialis…* rule in our system. Similar models of legal reasoning have also been applied in other computer science utilisations; for example, in [25] the authors are forced to implement one of the methods of resolving the conflicts between rules in multiagent systems.

Our model is based on proposition logic but there are some additions which allow for a better representation of specific features of the analysed problem. First of all, we introduce a distinction between two kinds of negation:* classical negation* (strong) and* negation as failure*. As negation as failure we understand the negation used in the conditional part of the rule. Such a negated condition is fulfilled if it is impossible to satisfy such a condition (it is false, it is not declared, or it is impossible to derive that it is satisfied). These two kinds of negation are used in a few logical systems, for example, in the Prakken and Sartor logic [26] or the Kowalski and Toni logic [27]. In our model the way of the utilisation of negation as failure is similar to the one presented by Prakken and Sartor in [26], but we do not allow to use construction like ~¬*P*, because in our model it is forbidden to use negation as failure in the consequence part of the rule and it is also forbidden to use classical negation in the antecedent part of the rule.

Another important point in our model, the conception of orders between facts, is based on a simplified version of the a'fortiori reasoning (reasoning from more to less: if norm N1 obliging to do more is binding, then norm N2 obliging to do less is binding more). In our work, this way of reasoning has been slightly modified: when condition *X* of a rule *r*_1_ is not satisfied literally, but there is a fact *Y* which satisfies this condition in a better way we may treat such a condition as satisfied. A more profound analysis and model of the a'fortiori reasoning can be found in [23].

The utilisation of defeasible implication in evaluation rules is another important feature of our model. The idea of distinction of two kinds of implication comes from the formal models of legal argumentation in which the problem of defeasibility of the rules is broadly discussed. In the Prakken and Sartor logic [26], all arguments are defeasible. Vreeswijk in his abstract argumentation system [28] uses two kinds of implication (material ⊃ and defeasible >). He assumes that the utilisation of defeasible implication requires the definition of a separate defeasible inference rule. Hage in his reason based logic [29] looks at the problem of defeasibility of the rules from another point of view, stating that this is a problem of the applicability of the rule. The rule could have satisfied conditions, but cannot be not applicable due to, for example, a conflict with another rule. Another interesting model of argumentation which uses defeasible and strict implication is introduced by Kowalski and Toni in [27]. In their system, each defeasible rule *r* has a condition ~defeated(*r*) (where ~ is negation as failure and defeated(*r*) is a predicate which denotes that this rule is defeated by another one) which allows defeating it if it remains in conflict with another rule *r*′ and has lower priority than *r*′.

Defeasible rules in our model are slightly different from those in the above mentioned models. The most important point in our model lies in the fact that only evaluation rules (which are not rules in a strict meaning of this word) are defeasible. Such a rule can be defeated only if its conditions are satisfied; it is in conflict with another rule with satisfied conditions and has a lower priority over the other one. Such a defeated rule is excluded from the reasoning process.

Torre and Tan in [30] define a few types of defeasibility and the one used in our model is closest to the* overridden defeasibility* which formalizes the cancellation of a rule by another one.

The ways of dealing with conflicts between rules and orders between these rules are discussed in the aforementioned works by Prakken and Sartor (i.e., [26, 31]) where the authors introduce their formal model of legal argumentation. The notion of conflict between rules in our approach is different from the one presented in their works, but the way of resolving it by the declaration of orders between rules and the assumption of defeasibility of rules is similar to the Prakken and Sartor model. In the above mentioned Kowalski and Toni logic, two rules are in conflict when they have complementary conclusions and the conditions of both rules are satisfied. Their model also uses two kinds of implication as well as two kinds of negation, but the way of dealing with conflict is slightly different from the one in our system. They define a few new predicates which are used to denote that the condition of the rule *r* is satisfied (holds(*r*)), rule *r* is defeated (defeated(*r*)) or two rules are in conflict (conflict(*r*, *r*′)).

## 4. Methodology of QoP Evaluation of Security Mechanisms

**Figure 1:**
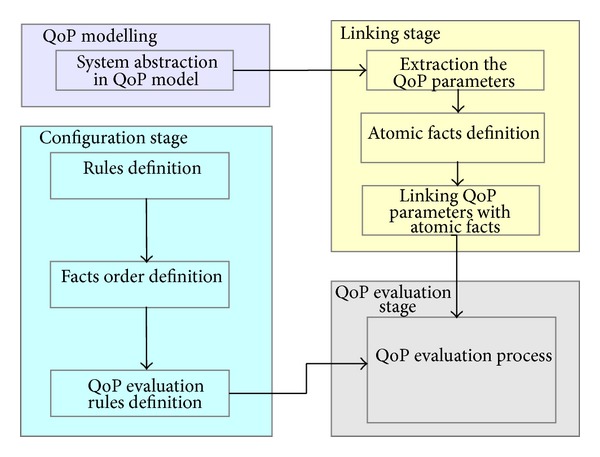
The methodology of QoP evaluation of security mechanisms.

The QoP evaluation of security mechanisms is a process which can be divided into four stages: QoP modelling, linking, configuration, and QoP evaluation. In Figure 1 the methodology of the process is presented.

*QoP Modelling Stage.* The first stage refers to modelling the system in one of the QoP models [4, 7–9, 12–17]. This phase depends on the chosen QoP approach, but the result should be the same, and the system must be modelled with QoP meaning (*system abstraction in the QoP model*). The proposed model can be used for different QoP modelling approaches.

*Linking Stage*. The next stage is responsible for the linking system modelled in the QoP model with the structure defined in the proposed model. Firstly, one has to extract all parameters used in the QoP model which refer to the factors which influence QoP parameters (*extraction of the QoP parameters*). After this, one creates atomic facts in the proposed model which are explicit to these extracted from the model (*atomic facts definition*). The parameters' names used in the QoP model can be different from those defined in the model, so one has to link them together (*linking QoP parameters with atomic facts*).

*Configuration Stage*. The next stage is the main phase where all structures proposed in the model for QoP evaluation of security mechanisms are defined. One can enumerate:* rules definition*,* facts order definition,* and* QoP evaluation rules definition*. All of these structures are described in detail in Section 2.

*QoP Evaluation Stage*. The last stage is responsible for QoP evaluation of security mechanisms. The analysed specification can be defined directly in the QoP model and thanks to the previously defined links between the QoP parameter and facts, a model of a case is generated. Finally, after the system version indication, the QoP evaluation of security mechanisms is performed (*QoP evaluation process*).

## 5. Case Study: TLS Handshake Protocol

**Figure 2:**
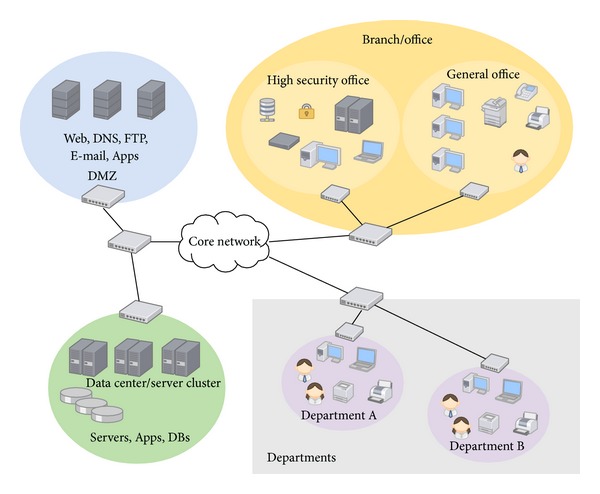
Example enterprise network architecture.

In this section we are going to present a case study of the QoP evaluation of security mechanisms for the TLS Handshake protocol. The TLS protocol is used each day in real business situations in the actual enterprise environment. Given the enterprise network infrastructure in Figure 2, one should analyse different roles which refer to different levels of the quality of protection of used security mechanisms. The users are allowed to access e-mail, FTP, web, and application servers with the communication channel protected by means of the TLS protocol at a different QoP level. The utilized versions of the TLS protocol together with equivalent cryptographic algorithms are summarized in Table 7.

The model for the TLS Handshake protocol can be found in the models library in the SMETool, which can be downloaded from the web page of the Quality of Protection Modelling Language Project [11]. In this case study, the client wants to verify the server and, next, to connect with the server by a secure connection. All of these security requirements can be realized by means of different security measures. We are analysing six versions of the protocol. The flow of the TLS Handshake protocol, which will be analysed further, is realized in five steps and the scheme is presented as shown in the following steps:

1. *C* → *S*:* ClientHello*
2. *S* → *C*:* ServerHello*,* Certificate*,* ServerKeyExchange*,* ServerHelloDone*
3. *C* → *S*:* ClientKeyExchange*,* ChangeCipherSpec*,* Finished*
4. *S* → *C*:* ChangeCipherSpec*,* Finished*
5. *C* → *S*:* Encrypted data.*

Below we present a description of these steps.

1. A Client sends the* ClientHello* message. This message contains the following attributes: the TLS Protocol version, session id, a list of available cipher suite, compression method, and random values.
2. The server responds with a* ServerHello*, which establishes the version of the TLS Protocol, session id (when session is resumed), cipher suite, and compression method. It also sends random values within* ServerHello*. Next the server sends the* Certificate* message which includes its certificate. Sending this message is not necessary; it depends on the selected cipher suite. The next message which may be sent is* ServerKeyExchange*. The server sends it when the server certificate is only for signing, or the server has no certificate. After this, the server sends* ServerHelloDone*, signalling that this phase is completed.
3. Next, the client sends the* ClientKeyExchange* message. Depending on the selected cipher, this message may have different contents. After this, the* ChangeCipherSpec* message is sent. The client sends it to signal the server that it has started to use the encryption. Finally, the encrypted* Finished* message is sent by the client.
4. Similarly, in response, the server sends* ChangeCipherSpec* and the encrypted* Finished* message.
5. The handshake is complete. Now, the server and client can exchange* Encrypted data*.

The methodology of the QoP evaluation of security mechanisms based on our model is presented in Section 3. In this section we have used the proposed methodology for analysing the TLS Handshake cryptographic protocol.

### 5.1. QoP Modelling

In the first step one has to model the system in one of the QoP models. In the presented case study, the TLS protocol is analysed as a full system. The QoP modelling process is a complex task so we are not going to present it in this paper. The example of the QoP model of TLS cryptographic protocol is presented in [6], where the protocol is modelled in the QoP-ML modelling language [12].

### 5.2. Linking Stage

#### 5.2.1. Extraction of the QoP Parameters

The first step in the linking stage refers to the extraction of all parameters used in the QoP model which refer to the factors which influence QoP. The form of the QoP parameters depends on the chosen QoP model. For example, in the QoP-ML modelling language, the system behaviour is changed by the functions which modify the states of the variables and pass the objects by communication channels. This structure is responsible for indicating the parameters which influence the system QoP.

Let us assume that *qp* is the QoP parameter in the QoP model which influences system security. The QoP parameter is atomic, which means that it cannot be split into other atomic QoP parameters. The *QP* is a set of the QoP parameters:

(12) $$\begin{array}{c} QP=\left\{qp_{1},qp_{2},qp_{3},\ldots ,qp_{n}\right\}, \end{array}$$

where

  
*qp*_1_, *qp*_2_, *qp*_3_,…, *qp*_*n*_are the QoP parameters which influence system security;
 
*QP* is a set of the QoP parameters.

#### 5.2.2. Atomic Facts Definition

In the next step, we declare a set of all possible facts *F* which will represent the features of the analysed systems.

In the presented case study we include the TLS cryptographic protocol. In the following section we define the atomic facts in the proposed model for the TLS protocol. These facts are taken from the official specification of the TLS protocol [32] where all possible versions of the protocol are defined. The facts are divided into three groups which refer to different factors: symmetric encryption (Table 4), message digest (Table 5), asymmetric encryption, and common facts (Table 6).

#### 5.2.3. Linking QoP Parameters with Atomic Facts

The names of the QoP parameters used in the QoP model can be different from atomic facts defined in the proposed model. In this step, the QoP parameters are linked with the facts in the proposed model in an explicit way.

**Definition 15 (linking operator).**

The linking operator ↦ denotes that one set of objects is mapped to another set of objects in an explicit way.

As we assume that the *QP* is the set of the QoP parameters used in the QoP model for the TLS protocol abstraction and *F* is the set of all atomic facts defined in the proposed model. Usually, the set of the QoP parameters *QP* is a subset of the facts in the model* F*, but in a special case this set can be equal to

(13) $$\begin{array}{c} QP=\left\{qp_{1},qp_{2},qp_{3},\ldots ,qp_{n}\right\};\\ F=\left\{f_{1},f_{2},f_{3},\ldots ,f_{n}\right\};\\ Z\in F, \end{array}$$

where

 
*qp*_1_, *qp*_2_, *qp*_3_,…, *qp*_*n*_ are QoP parameters which influence the system security;
 
*QP* is the set of QoP parameters;
 
*f*_1_, *f*_2_, *f*_3_,…, *f*_*n*_ are facts in the formal model;
 
*F* is the set of all facts in the model;
 
*Z* is the subset of all facts in the model.

QoP parameters from the QoP model are linked with the subset of the facts defined in the model in an explicit way by the linking operator ↦:

(14) $$\begin{array}{c} QP⟼Z. \end{array}$$

### 5.3. Configuration Stage

The next stage is the main phase where all structures proposed in the model for the QoP evaluation of security mechanisms are defined. Among them, one can enumerate definitions of rules, facts order, and the QoP evaluation rules.

#### 5.3.1. Rules Definition

Based on the TLS cryptographic protocol specification [32], we specify the rules which refer to a possible realization of the protocol. In Appendix A one can find the rules defined for the TLS protocol.

#### 5.3.2. Facts Order Definition

In the next step, the facts order must be defined. The order between facts can be defined only for the same security attribute (SA). For the presented example, we evaluate the QoP of security mechanisms in the case of four security attributes: integrity (I), confidentiality (C), authentication (Au), and availability (A). We define the facts order according to the expert knowledge in the field of cryptographic protocols. All the defined fact orders are presented in Appendix B.

#### 5.3.3. QoP Evaluation Rules Definition

The last step in the configuration stage is to define the QoP evaluation rules. For the TLS cryptographic protocol, the evaluation rules refer to the same four security attributes: integrity (I), confidentiality (C), authentication (Au), and availability (A). According to these rules, the QoP evaluation of security mechanism of the TLS protocol is performed. In the literature, defining the influence of specific security mechanisms is based on expert knowledge in the field of cryptology and system security [7, 8]. In our example, we perform the same expert knowledge analysis which refers to the TLS cryptographic protocol. The defined QoP evaluation rules are presented in Appendix C.

### 5.4. QoP Evaluation Stage

Having finished the configuration stage, one can start the QoP evaluation process. In the presented example we choose six versions of the TLS protocol which are presented in Table 7. These cipher suites are described in detail in the TLS specification [32]. The fifth and sixth versions are modified according to the versions presented in the TLS specification. In the fifth version we analyse a case where a new cryptographic module, compared to the previously defined ones, will be possible. This module is the implementation of HMAC-SHA512 [33]. The sixth version is identical with the first one except that compression is enabled.

The first version is the most popular cipher suite for online banking. Some banks operate their online services using the second TLS protocol version. The third version can be used when authorisation is not required. The fourth version accomplishes the TLS protocol with the strongest set of security parameters. The fifth version analyses the hypothetical scenario when a new implementation of one of the cryptographic modules is possible. The sixth version is identical with the first one, but the data are compressed before encryption.

These six versions are analysed as 6 cases which represent 6 different realizations of the TLS protocol. These versions can be represented as the following set of facts.

**Case 1 .**

*C*_1_ = {*f*_1_(PK), *f*_1_(cipher), *f*_1_(mac), *f*_1_(key)}.

**Case 2 .**

*C*_2_ = {*f*_3_(cipher), *f*_1_(key), *f*_1_(*mode*), *f*_2_(mac), *f*_1_(PK)}.

**Case 3 .**

*C*_3_ = {*f*_1_(cipher), *f*_6_(PK), *f*_1_(key), *f*_1_(mac)}.

**Case 4 .**

*C*_4_ = {*f*_5_(PK), *f*_3_(cipher), *f*_3_(key), *f*_1_(*mode*), *f*_3_(mac)}.

**Case 5 .**

*C*_5_ = {*f*_1_(PK), *f*_1_(cipher), *f*_4_(mac), *f*_1_(key)}.

**Case 6 .**

*C*_6_ = {*f*_1_(PK), *f*_1_(cipher), *f*_1_(mac), *f*_1_(key), *f*_1_(*com*)}.

In Case 5, one can find the fact *f*_4_(mac), which is not defined in Table 5. The example is a situation in which a new protocol implementation will be realized and the QoP evaluation system does not provide analysis for this version. In such a case, this fact will be added later during the detailed analysis of this case.

After defining the facts which describe the analysed cases (*C*), one has to derive (⊢) other atomic or complex facts (*C*′): *C*⊢*C*′. They can be derived by using the earlier described inference mechanism and the rules defined in Appendix A. On the basis of this knowledge, one can prepare final evaluation of security mechanisms represented as the security attributes. Below we present the evaluation of the six analysed cases.

*QoP Evaluation of Case 1*.

Consider the following:

(15) C1⊢C1′;C1′={f1(mac−len),f1(k−len),¬f1(IV),¬f2(IV),¬f1(bs),¬f2(bs),f(mac−len),f(k−len),f(PK),f(cipher),f(mac),f(key),¬f2(PK),¬f3(PK),¬f4(PK),¬f5(PK),¬f6(PK),¬f2(cipher),¬f3(cipher),¬f2(mac),¬f3(mac),¬f2(key),¬f3(key),¬f4(key),¬f2(mac−len), ¬f3(mac−len),¬f2(k−len),¬f3(k−len)}.

Case 1 is denoted as *P*_1_ and is a union of sets *C*_1_ and *C*_1_′:

(16) $$\begin{array}{c} P_{1}=\left(C_{1}\cup C_{1}^{\prime }\right). \end{array}$$

The QoP evaluation of the security attributes

(17) O1={〈confidentiality,2〉,〈integrity,3〉,〈availability,5〉,〈authorisation,1〉}.

*QoP Evaluation of Case 2*.

Consider the following:

(18) C2⊢C2′;C2′={f2(mac−len),f2(k−len),f2(IV),f2(bs),f2(CS),f(cipher),f(key),f(mac),f(PK),f(mac−len),f(k−len),f(IV),f(bs),¬f2(PK),¬f3(PK),¬f4(PK),¬f5(PK),¬f6(PK),¬f2(cipher),¬f1(cipher),¬f1(mac),¬f3(mac),¬f2(key),¬f3(key),¬f4(key),¬f1(mac−len),¬f3(mac−len),¬f1(k−len),¬f3(k−len),¬f1(bs),¬f1(IV)}.

Case 2 is denoted as *P*_2_ and is a union of sets *C*_2_ and *C*_2_′:

(19) $$\begin{array}{c} P_{2}=\left(C_{2}\cup C_{2}^{\prime }\right). \end{array}$$

The QoP evaluation of the security attributes

(20) O2={〈confidentiality,8〉,〈integrity,6〉,〈availability,10〉,〈authorisation,1〉}.

*QoP Evaluation of Case 3*.

Consider the following:

(21) C3⊢C3′;C3′={f1(mac−len),f1(k−len),¬f1(IV),¬f2(IV),¬f1(bs),¬f2(bs),f(mac−len),f(k−len),f(PK),f(cipher),f(mac),f(key),¬f2(PK),¬f3(PK),¬f4(PK),¬f5(PK),¬f1(PK),¬f2(cipher),¬f3(cipher),¬f2(mac),¬f3(mac),¬f2(key),¬f3(key),¬f4(key),¬f2(mac−len),¬f3(mac−len),¬f2(k−len),¬f3(k−len)}.

Case 3 is denoted as *P*_3_ and is a union of sets *C*_3_ and *C*_3_′:

(22) $$\begin{array}{c} P_{3}=\left(C_{3}\cup C_{3}^{\prime }\right). \end{array}$$

The QoP evaluation of the security attributes

(23) O3={〈confidentiality,2〉,〈integrity,3〉,〈availability,5〉,〈authorisation,0〉}.

*QoP Evaluation of Case 4*.

Consider the following:

(24) C4⊢C4′;C4′={f3(mac−len),f3(k−len),f2(IV),f2(bs),f2(CS),f(PK),f(cipher),f(key),f(mode),f(mac),f(mac−len),f(k−len),f(IV),f(bs),¬f2(PK),¬f3(PK),¬f4(PK),¬f6(PK),¬f1(PK),¬f1(cipher),¬f2(cipher),¬f1(mac),¬f2(mac),¬f1(mac−len),¬f2(mac−len),¬f1(k−len),¬f2(k−len),¬f1(IV),¬f1(bs)}.

Case 4 is denoted as *P*_4_ and is a union of sets *C*_4_ and *C*_4_′:

(25) $$\begin{array}{c} P_{4}=\left(C_{4}\cup C_{4}^{\prime }\right). \end{array}$$

The QoP evaluation of the security attributes

(26) O4={〈confidentiality,10〉,〈integrity,9〉,〈availability,15〉,〈authorisation,3〉}.

*QoP Evaluation of Case 5*. Case 5 is different from the previous four cases because there is a fact declared (*f*_4_(mac)) which is not in the conditional part of any rule. As we have stated before, a declaration of order between facts is much easier than adding new rules. Based on that, we declare two new orders:

(27) $$\begin{array}{c} f_{4}\left(\text{mac}\right){>}_{I}f_{3}\left(\text{mac}\right){>}_{I}f_{2}\left(\text{mac}\right){>}_{I}f_{1}\left(\text{mac}\right);\\ f_{1}\left(\text{mac}\right){>}_{A}f_{2}\left(\text{mac}\right){>}_{A}f_{3}\left(\text{mac}\right){>}_{A}f_{4}\left(\text{mac}\right). \end{array}$$

These orders mean that *HMAC* − *SHA*512 is more than *HMAC* − *SHA*256 in the context of integrity, but it is also less than *HMAC* − *SHA*256 in the context of availability.

Adding these orders to the knowledge base results in satisfying conditions of all rules which have *f*_3_(mac) in their conditional parts:

(28) C5⊢C5′;C5′={f3(mac−len),f3(k−len),¬f1(IV),¬f2(IV),¬f1(bs),¬f2(bs),f(mac−len),f(k−len),f(PK),f(cipher),f(mac),f(key),¬f2(PK),¬f3(PK),¬f4(PK),¬f1(mac),¬f5(PK),¬f6(PK),¬f2(cipher),¬f3(cipher),¬f2(mac),f3(mac),¬f2(key),¬f3(key),¬f4(key),¬f2(mac−len),¬f1(mac−len),¬f2(k−len),¬f1(k−len)}.

Case 5 is denoted as *P*_5_ and is a union of sets *C*_5_ and *C*_5_′:

(29) $$\begin{array}{c} P_{5}=\left(C_{5}\cup C_{5}^{\prime }\right). \end{array}$$

The QoP evaluation of the security attributes

(30) O5={〈confidentiality,2〉,〈integrity,9〉,〈availability,11〉,〈authorisation,1〉}.

*QoP Evaluation of Case 6.* Case 6 is the same as Case 1 with the difference that the compression is enabled:

(31) C6⊢C6′;C6′={f1(mac−len),f1(k−len),¬f1(IV),¬f2(IV),¬f1(bs),¬f2(bs),f(mac−len),f(k−len),f(PK),f(cipher),f(mac),f(key),¬f2(PK),¬f3(PK),¬f4(PK),¬f5(PK),¬f6(PK),¬f2(cipher),¬f3(cipher),¬f2(mac),¬f3(mac),¬f2(key),¬f3(key),¬f4(key),¬f2(mac−len),¬f3(mac−len),¬f2(k−len),¬f3(k−len)}.

Case 6 is denoted as *P*_6_ and is a union of sets *C*_6_ and *C*_6_′:

(32) $$\begin{array}{c} P_{6}=\left(C_{6}\cup C_{6}^{\prime }\right). \end{array}$$

This case presents the situation in which we have two conflicting evaluation rules:

(33) f1(cipher)⟹In⁡f1(A) which  we  denote  as  er1,f1(COM)∧f1(cipher)⟹In⁡f4(A),which  we  denote  as  er36.

The rule *er*_36_ is subsumed by the rule *er*_1_ because every case which satisfies the rule *er*_36_ also satisfies the rule *er*_1_. Both of these rules evaluate the same security attribute and both of them, in our case, satisfy the conditions. Based on the previously defined mechanism of recognition of subsuming and conflicting rules, we will treat these rules as conflicting ones and the rule *er*_36_ will defeat the rule *er*_1_. The QoP evaluation of the security attributes

(34) O6={〈confidentiality,2〉,〈integrity,3〉,〈availability,8〉,〈authorisation,1〉}.

The results obtained by the QoP evaluation of security mechanisms are presented in Table 8. These results are quantitative.

#### 5.4.1. Qualitative Estimation

The results are presented as quantitative estimation of security attributes. During the QoP evaluation of security mechanisms one can introduce qualitative interpretation of the results. That kind of estimation is made for the all security attributes.

In the presented example, we introduce 5 levels of evaluation: very low, low, medium, high, and very high. It is important that the existing correlations between the quantitative and qualitative results have not only a theoretical character but also a real one. A practical character of the qualitative estimation of the security attributes is obtained because the minimal and maximal possible values of the security attributes for a particular version of the analyzed protocol are calculated. The ranges of parameters for the qualitative evaluation are calculated by formula (35).

For the analysed versions of the TLS protocol, the qualitative assessment will be prepared according to the ranges presented in Table 9. These ranges are calculated according to formula (35). On the basis of the calculated ranges, the qualitative assessment of the TLS protocol is obtained. These marks are presented in Table 10:

(35) very  low=(Qmin⁡,Qmin⁡+X〉;low=(Qmin⁡+X,Qmin⁡+2X〉;medium=(Qmin⁡+2X,Qmin⁡+3X〉;high=(Qmin⁡+3X,Qmin⁡+4X〉;very  high=(Qmin⁡+4X,Qmin⁡+5X〉,whereX=Qmax⁡−Qmin⁡5,

where

 
*Q*_max⁡_ is the maximum value for the security attribute among all analysed versions of the protocol;
 
*Q*_min⁡_ is the minimum value for the security attribute among all analysed versions of the protocol.

After the QoP evaluation of security mechanisms one can interpret the results. The first and third versions of the TLS protocol are the most efficient ones in the case of CPU performance. This fact is indicated by the availability attribute. This is due to the fact that the applied security mechanisms are the most efficient ones. However, the analysis of confidentiality and integrity indicates that these attributes are accomplished on the lowest level of all analysed versions.

One of the main functions of the TLS protocol is server authorisation. Versions 1, 3, 5, and 6 guarantee this attribute on the lowest level, but the third version does not guarantee it at all. The fourth protocol version achieves the strongest possible security level. This fact influences the system performance which is indicated by the availability attribute.

In a certain scenario one can use the TLS protocol version which is between the strongest and poorest security levels (*medium*). Then one can use the second version. This version guarantees the confidentiality on the high level and the integrity on the medium level. The authorisation is still guaranteed, but on the very low level. The set of parameters used in the second version allows the decrease of the system performance according to the strongest fourth version to the medium level.

The fifth and sixth versions should be compared with reference to the first version because they are a modification of the first version. In the fifth version, the new cryptographic module which guarantees the integrity on the strongest possible level is introduced. Increasing the level of protection results in a decreased performance, which is indicated by the medium level of the availability attribute. In the sixth version, the compression is enabled which decreases the system performance in comparison to the first version.

### 5.5. Formal Model Goals Evaluation

At the beginning of the formal model definition we enumerated the goals of our model. In this section, we would like to present the goals achieved by our model.

(1)* Goal 1: Automatic QoP Evaluation of the Not Directly Defined Scenarios*. One of the most important features of the model is the QoP security evaluation based on the not directly defined scenarios. It means that during the analysis, one can prepare the QoP evaluation of one of the cryptographic protocol versions which is not directly defined. In our model the inference mechanism and rules are defined and owing to that, one can automatically derive the set of facts which describes the analysed version of the protocol. Since our model is based on the analysis of basic mechanisms of security protection and relations between them, the evaluation process does not require an advance preparation of direct scenarios describing all possible configurations of analysed systems. In our model the mechanism which allows for the recognition and resolution of conflicts between evaluation rules is introduced.

(2)* Goal 2: Quality of Protection Evaluation of All Security Mechanisms. *In the proposed model one can evaluate any of the security mechanisms with regard to the quality of protection factor. The security mechanisms, modelled in one of the QoP models, are mapped to the atomic or complex facts. In our model, one can define the facts that any of the security mechanisms can be represented by. The linking stage described in the methodology illustrates the mapping process. Finally, on the basis of the QoP evaluation rules and their order one can prepare the QoP evaluation of security mechanisms.

(3)* Goal 3: Analysis Refers to All Security Attributes*. The security attribute describes system behaviour in terms of information security requirements. That kind of behaviour is changed by the security mechanisms which modify the modelled system. As we mentioned above, in our model one can represent any security mechanism and analyse its influence on any security attribute.

(4)* Goal 4: The model Can Be Used for any QoP Models*. The presented model can be applied to the systems which are modelled by any of the QoP models. This goal is achieved due to the* linking operator* which links the QoP parameters from the QoP model with the subset of facts defined in the model. These objects are mapped in an explicit way.

### 5.6. Comparison of Our Model with Existing Approaches

In the literature one can find different approaches [4, 7–9] dealing with the quality of protection evaluation of security mechanisms. In Table 11 we compare the model presented in this paper with the existing approaches. These approaches can be characterized by the following main attributes.

 
*Quantitative assessment* refers to the quantitative assessment of the estimated quality of protection. All of the presented approaches, except one, allow quantitative assessment of the QoP evaluation of security mechanisms. Petriu et al. [9] discuss performance analysis in terms of the used security level, but the analysis has an qualitative character.
 
*Formal representation* refers to the representation of the quality of protection evaluation of security mechanisms by mathematical formulae. Among the enumerated approaches only the one presented in this paper has formal representation, while the others are represented by means of an analytical model, without any formal definition of the objects and rules required for formal evaluation.
 
*Executability* specifies the possibility of the implementation of an automated tool able to perform the evaluation of QoP mechanisms. The tool support is provided for three approaches: Ksiezopolski and Kotulski [4] model is supported by the SPOT tool, Petriu et al. [9] create the UMLsec tool, and the presented model is supported by the SME tool, which can be downloaded from [11].
 
*Indirect reasoning* reasoning for not directly defined scenarios. All of the presented approaches, except the new model presented in this paper, have one significant limitation. These models can evaluate only these versions which were previously directly defined and described in detail. The presented model provides the method for indirect reasoning.
 
*Holistic* is the possibility of the evaluation of all security attributes. All presented models, except one, can be used for the evaluation of all security attributes. Only the model presented by Petriu et al. [9] focuses on performance analysis and is related to availability.
 
*Completeness* is the possibility of the representation of all security mechanisms. This attribute is provided for all models.

## 6. Conclusions

In the paper we propose a formal model for the quality of protection evaluation of security mechanisms. The presented method has four main features. Firstly, the presented approach allows for the QoP evaluation for the system for which the scenarios of assessment of security mechanisms are not directly defined. Secondly, all security mechanisms can be analysed with regard to the QoP factor. Thirdly, analysis can be performed for all security attributes. Finally, the proposed model can be used for any QoP models.

Our method also has some interesting features which make the evaluation of a system easier. A system can be incrementally developed by adding new knowledge. To avoid conflicts between new and old rules, our model includes the mechanism of recognition and resolution of a specific kind of conflict between evaluation rules.

In the paper we have presented the methodology of our model's preparation. On the basis of this methodology, we have created the model of the TLS cryptographic protocol. We have also performed the QoP evaluation of the sixth selected version of the TLS protocol. The main analysis refers to the quantitative assessment of four security attributes: confidentiality, integrity, availability, and authorisation. Finally, we have introduced the formula for qualitative interpretation of the prepared QoP evaluation.

An additional contribution of the paper is the implementation of the security mechanisms evaluation tool (SMETool) which supports the presented method. The SMETool can be downloaded from the web page of the Quality of Protection Modelling Language Project [11]. The analysed model for the TLS Handshake protocol can be found in the models library in the SMETool.

## Figures and Tables

**Algorithm 1:**
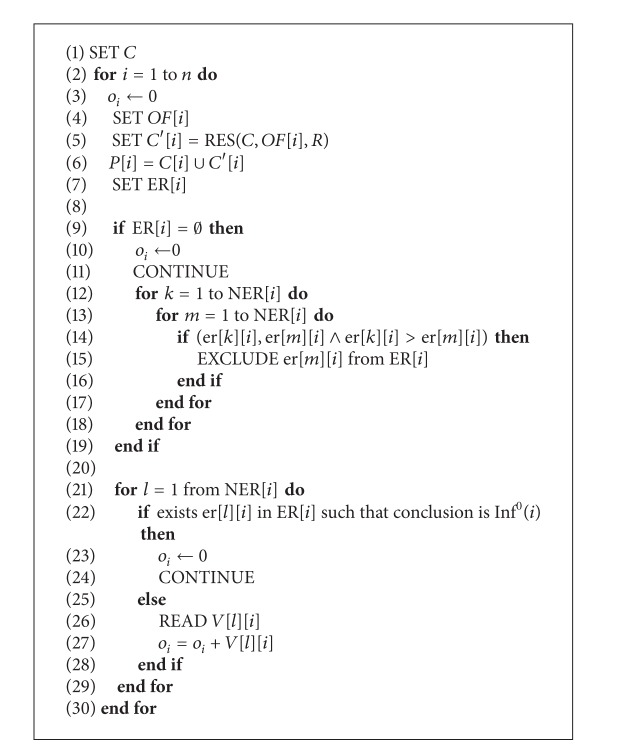
Algorithm of security attributes evaluation.

**Table 1:** Selection of the specific cryptographic algorithms.

***Ciphers***	

*Version 1—low *	

**RC2-CBC + MD5 **	

*Version 2—medium *	

** DES-CBC + SHA1 **	

*Version 3—high *	

**3DES-CBC + SHA1 **	

**Table 2:** The bit rate of VPN connections.

*Version 1—low *	

Bit rate	501 KB/s

*Version 2—medium *	

Bit rate	480 KB/s

*Version 3—high *	

Bit rate	453 KB/s

**Table 3:** The parameters and variables for the security attributes evaluation algorithm.

SET	Make a choice indication
EXCLUDE	Excluding from the ER indication
READ	Reading indication
CONTINUE	Processing statement will be skipped
RES(*C*, *OF*[*i*], *R*)	The reasoning function based on a set of facts *C* and order of facts *OF*[*i*] for the security attribute *i* and rules *R* (inference mechanisms)
*OF*[*i*]	Orders between facts referred to a security attribute *i*
*C*	A case expressed by a set of facts
*C*′	A set of facts obtained from the inference mechanism
*P*[*i*]	A full description of a case for a security attribute *i*
*R*	A set of rules
*k*, *m*, *l*	Indicates the current evaluation rule
*o*_*i*_	The evaluation of *i*th security attribute
ER[*i*]	A set of evaluation rules with satisfied conditions for the security attribute *i*
NER[*i*]	The number of rules with satisfied conditions for the security attribute *i*
er[*x*][*i*]	The evaluation rule *x* for the security attribute *i*
*i*	The index of the current security attribute
*n*	The quantity of security attributes
*V*[*l*][*i*]	The value of the security influence of the security mechanisms represented by the evaluation rule *l* for the security attribute *i*

**Table 4:** The group of facts refers to symmetric encryption.

Facts	
Group name: cipher (symmetric encryption algorithm)	
*f*_1_(cipher) = RC4	
*f*_2_(cipher) = 3DES	
*f*_3_(cipher) = AES	
Group name: bs (block size in bytes)	
*f*_1_(bs) = 8	
*f*_1_(bs) = 16	
Group name: IV (initiate vector in bytes)	
*f*_1_(IV) = 8	
*f*_1_(IV) = 16	
Group name: key (key length in bytes)	
*f*_1_(key) = 16	
*f*_2_(key) = 24	
*f*_3_(key) = 32	

**Table 5:** The group of facts refers to message digest.

Facts	
Group name: mac (message authentication code algorithm)	
f_1_(mac) = HMAC-MD5	
f_2_(mac) = HMAC-SHA1	
f_3_(mac) = HMAC-SHA256	
Group name: mac-len (message digest length in bytes)	
f_1_(mac-len) = 16	
f_2_(mac-len) = 20	
f_3_(mac-len) = 32	
Group name: k-len (mac key length in bytes)	
f_1_(k-len) = 16	
f_2_(k-len) = 20	
f_3_(k-len) = 32	

**Table 6:** The group of facts refers to asymmetric cryptography and common facts.

Facts	
Group name: PK (key exchange algorithm scheme)	
*f*_1_(PK) = RSA	
*f*_2_(PK) = DH-DSS	
*f*_3_(PK) = DH-RSA	
*f*_4_(PK) = DHE-DSS	
*f*_5_(PK) = DHE-RSA	
*f*_6_(PK) = DH-anon	
Group name: mode (mode of operation)	
*f*_1_(mode) = CBC	
Group name: com (bit compression before encryption)	
*f*_1_(com) = Compression	

**Table 7:** The analysed versions of TLS protocol.

Version	The cipher suite
1	TLS_RSA_WITH_RC4_128_MD5
2	TLS_RSA_WITH_AES_128_CBC_SHA
3	TLS_DH_anon_WITH_RC4_128_MD5
4	TLS_DHE_RSA_WITH_AES_256_CBC_SHA256
5	TLS_RSA_WITH_RC4_128_newSHA512
6	TLS_RSA_WITH_RC4_128_MD5 + COM

**Table 8:** The QoP evaluation of the analysed versions of TLS protocol.

Version	*C*	*I*	*A*	Au
1	2	3	5	1
2	8	6	10	1
3	2	3	5	0
4	10	9	15	3
5	2	9	11	1
6	2	3	8	1

**Table 9:** The ranges for the qualitative interpretation of QoP evaluation of the analysed versions of TLS protocol.

Mark	*C*	*I*	*A*	Au
Very low	(2,3.6〉	(3,4.2〉	(5,7〉	(1,1.4〉
Low	(3.6,5.2〉	(4.2,5.4〉	(7,9〉	(1.4,1.8〉
Medium	(5.2,6.8〉	(5.4,6.6〉	(9,11〉	(1.8,2.2〉
High	(6.8,8.4〉	(6.6,7.8〉	(11,13〉	(2.2,2.6〉
Very high	(8.4,10〉	(7.8,9〉	(13,15〉	(2.6,3〉

**Table 10:** The qualitative interpretation of QoP evaluation of the analysed versions of TLS protocol.

Version	*C*	*I*	*A*	Au
1	Very low	Very low	Very low	Very low
2	High	Medium	Medium	Very low
3	Very low	Very low	Very low	No
4	Very high	Very high	Very high	Very high
5	Very low	Very high	Medium	Very low
6	Very low	Very low	Low	Very low

**Table 11:** The characterization of the security mechanisms evaluation models.

	Agarwal and Wang [7]	Ksiezopolski and Kotulski [4]	Luo et al. [8]	Petriu et al. [9]	Our model
Quantitative assessment	√	√	√	—	√
Formal representation	—	—	—	—	√
Executability	—	√	—	√	√
Indirect reasoning	—	—	—	—	√
Holistic	√	√	√	—	√
Completeness	√	√	√	√	√

## Appendices

### A. The Rules Definition for TLS Cryptographic Protocol

The fact based rules are as follows:

*f*_1_(cipher)∨*f*_2_(cipher)∨*f*_3_(cipher) → *f*(cipher)
*f*_1_(*bs*)∨*f*_2_(*bs*) → *f*(*bs*)
*f*_1_(*IV*)∨*f*_2_(*IV*) → *f*(*IV*)
*f*_1_(key)∨*f*_2_(key)∨*f*_3_(key) → *f*(key)
*f*_1_(mac)∨*f*_2_(mac)∨*f*_3_(mac) → *f*(mac)
*f*_1_(mac − len)∨*f*_2_(mac − len)∨*f*_3_(mac − len) → *f*(mac − len)
*f*_1_(*k* − len)∨*f*_2_(*k* − len)∨*f*_3_(*k* − len) → *f*(*k* − len)
*f*_1_(PK)∨*f*_2_(PK)∨*f*_3_(PK)∨*f*_4_(PK)∨*f*_5_(PK)∨*f*_6_(PK) → *f*(PK)
~*f*(cipher)→¬*f*_1_(key)∧¬*f*_2_(key)∧¬*f*_3_(key)∧¬*f*_1_(*bs*)∧¬*f*_2_(*bs*)∧¬*f*_1_(*IV*)∧¬*f*_2_(*IV*)
~*f*(mac)→¬*f*(mac − len)∧¬*f*(*k* − len)
*f*_1_(mac) → *f*_1_(mac − len)∧*f*_1_(*k* − len)
*f*_2_(mac) → *f*_2_(mac − len)∧*f*_2_(*k* − len)
*f*_3_(mac) → *f*_3_(mac − len)∧*f*_3_(*k* − len)
*f*_1_(cipher) → *f*_1_(key)∧¬*f*_1_(*IV*)∧¬*f*_2_(*IV*)∧¬*f*_1_(*bs*)∧¬*f*_2_(*bs*)
*f*_2_(cipher) → *f*_2_(key)∧*f*_1_(*IV*)∧*f*_1_(*bs*)
*f*_3_(cipher) → *f*_2_(*IV*)∧*f*_2_(*bs*).

The rules which define the exclusive facts are as follows:

*f*_1_(cipher)→¬*f*_2_(cipher)∧¬*f*_3_(cipher)
*f*_2_(cipher)→¬*f*_1_(cipher)∧¬*f*_3_(cipher)
*f*_3_(cipher)→¬*f*_2_(cipher)∧¬*f*_1_(cipher)
*f*_1_(*bs*)→¬*f*_2_(*bs*)
*f*_2_(*bs*)→¬*f*_1_(*bs*)
*f*_1_(*IV*)→¬*f*_2_(*IV*)
*f*_2_(*IV*)→¬*f*_1_(*IV*)
*f*_1_(key)→¬*f*_2_(key)∧¬*f*_3_(key)∧¬*f*_4_(key)
*f*_2_(key)→¬*f*_1_(key)∧¬*f*_3_(key)∧¬*f*_4_(key)
*f*_3_(key)→¬*f*_2_(key)∧¬*f*_1_(key)∧¬*f*_4_(key)
*f*_4_(key)→¬*f*_2_(key)∧¬*f*_3_(key)∧¬*f*_1_(key)
*f*_1_(mac)→¬*f*_2_(mac)∧¬*f*_3_(mac)
*f*_2_(mac)→¬*f*_1_(mac)∧¬*f*_3_(mac)
*f*_3_(mac)→¬*f*_2_(mac)∧¬*f*_1_(mac)
*f*_1_(mac − len)→¬*f*_2_(mac − len)∧¬*f*_3_(mac − len)
*f*_2_(mac − len)→¬*f*_1_(mac − len)∧¬*f*_3_(mac − len)
*f*_3_(mac − len)→¬*f*_2_(mac − len)∧¬*f*_1_(mac − len)
*f*_1_(*k* − len)→¬*f*_2_(*k* − len)∧¬*f*_3_(*k* − len)
*f*_2_(*k* − len)→¬*f*_1_(*k* − len)∧¬*f*_3_(*k* − len)
*f*_3_(*k* − len)→¬*f*_2_(*k* − len)∧¬*f*_1_(*k* − len)
*f*_1_(PK)→¬*f*_2_(PK)∧¬*f*_3_(PK)∧¬*f*_4_(PK)∧¬*f*_5_(PK)∧¬*f*_6_(PK)
*f*_2_(PK)→¬*f*_1_(PK)∧¬*f*_3_(PK)∧¬*f*_4_(PK)∧¬*f*_5_(PK)∧¬*f*_6_(PK)
*f*_3_(PK)→¬*f*_2_(PK)∧¬*f*_1_(PK)∧¬*f*_4_(PK)∧¬*f*_5_(PK)∧¬*f*_6_(PK)
*f*_4_(PK)→¬*f*_2_(PK)∧¬*f*_3_(PK)∧¬*f*_1_(PK)∧¬*f*_5_(PK)∧¬*f*_6_(PK)
*f*_5_(PK)→¬*f*_2_(PK)∧¬*f*_3_(PK)∧¬*f*_4_(PK)∧¬*f*_1_(PK)∧¬*f*_6_(PK)
*f*_6_(PK)→¬*f*_2_(PK)∧¬*f*_3_(PK)∧¬*f*_4_(PK)∧¬*f*_5_(PK)∧¬*f*_1_(PK).

### B. The Facts Order Definition for the TLS Cryptographic Protocol

Orders between facts are as follows:

*f*_3_(cipher)>_*C*_ *f*_2_(cipher)>_*C*_ *f*_1_(cipher)
*f*_1_(cipher)>_*A*_ *f*_2_(cipher)>_*A*_ *f*_3_(cipher)
*f*_2_(*bs*)>_*C*_ *f*_1_(*bs*)
*f*_2_(*IV*)>_*C*_ *f*_1_(*IV*)
*f*_3_(key)>_*C*_ *f*_2_(key)>_*C*_ *f*_1_(key)
*f*_1_(key)>_*A*_ *f*_2_(key)>_*A*_ *f*_3_(key)
*f*_3_(mac)>_*I*_ *f*_2_(mac)>_*I*_ *f*_1_(mac)
*f*_1_(mac)>_*A*_ *f*_2_(mac)>_*A*_ *f*_3_(mac)
*f*_3_(mac − len)>_*I*_ *f*_2_(mac − len)>_*I*_ *f*_1_(mac − len)
*f*_1_(mac − len)>_*A*_ *f*_2_(mac − len)>_*A*_ *f*_3_(mac − len)
*f*_3_(*k* − len)>_*I*_ *f*_2_(*k* − len)>_*I*_ *f*_1_(*k* − len)
*f*_1_(*k* − len)>_*A*_ *f*_2_(*k* − len)>_*A*_ *f*_3_(*k* − len)
*f*_2_(PK)>_*AU*_ *f*_1_(PK)
*f*_3_(PK)>_*AU*_ *f*_1_(PK)
*f*_4_(PK)>_*AU*_ *f*_2_(PK)
*f*_5_(PK)>_*AU*_ *f*_2_(PK)
*f*_4_(PK)>_*AU*_ *f*_3_(PK)
*f*_5_(PK)>_*AU*_ *f*_3_(PK)
*f*_1_(PK)>_*A*_ *f*_2_(PK)
*f*_1_(PK)>_*A*_ *f*_3_(PK)
*f*_2_(PK)>_*A*_ *f*_4_(PK)
*f*_2_(PK)>_*A*_ *f*_5_(PK)
*f*_3_(PK)>_*A*_ *f*_4_(PK)
*f*_3_(PK)>_*A*_ *f*_5_(PK).

### C. The QoP Evaluation Rules Definition for the TLS Cryptographic Protocol

The evaluation rules are as follows:

*f*_1_(cipher)⇒In⁡f^1^(*A*)
*f*_2_(cipher)⇒In⁡f^2^(*A*)
*f*_3_(cipher)⇒In⁡f^3^(*A*)
*f*_1_(cipher)⇒In⁡f^1^(*C*)
*f*_2_(cipher)⇒In⁡f^2^(*C*)
*f*_3_(cipher)⇒In⁡f^3^(*C*)
*f*_1_(*bs*)⇒In⁡f^1^(*C*)
*f*_2_(*bs*)⇒In⁡f^2^(*C*)
*f*_1_(*IV*)⇒In⁡f^1^(*C*)
*f*_2_(*IV*)⇒In⁡f^2^(*C*)
*f*_1_(key)⇒In⁡f^1^(*C*)
*f*_2_(key)⇒In⁡f^2^(*C*)
*f*_3_(key)⇒In⁡f^3^(*C*)
*f*_1_(key)⇒In⁡f^1^(*A*)
*f*_2_(key)⇒In⁡f^2^(*A*)
*f*_3_(key)⇒In⁡f^3^(*A*)
*f*_1_(mac)⇒In⁡f^1^(*I*)
*f*_2_(mac)⇒In⁡f^2^(*I*)
*f*_3_(mac)⇒In⁡f^3^(*I*)
*f*_1_(mac)⇒In⁡f^1^(*A*)
*f*_2_(mac)⇒In⁡f^2^(*A*)
*f*_3_(mac)⇒In⁡f^3^(*A*)
*f*_1_(mac − len)⇒In⁡f^1^(*I*)
*f*_2_(mac − len)⇒In⁡f^2^(*I*)
*f*_3_(mac − len)⇒In⁡f^3^(*I*)
*f*_1_(mac − len)⇒In⁡f^1^(*A*)
*f*_2_(mac − len)⇒In⁡f^2^(*A*)
*f*_3_(mac − len)⇒In⁡f^3^(*A*)
*f*_1_(*k* − len)⇒In⁡f^1^(*I*)
*f*_2_(*k* − len)⇒In⁡f^2^(*I*)
*f*_3_(*k* − len)⇒In⁡f^3^(*I*)
*f*_1_(*k* − len)⇒In⁡f^1^(*A*)
*f*_2_(*k* − len)⇒In⁡f^2^(*A*)
*f*_3_(*k* − len)⇒In⁡f^3^(*A*)
*f*_1_(PK)⇒In⁡f^1^(*Au*)
*f*_2_(PK)⇒In⁡f^2^(*Au*)
*f*_3_(PK)⇒In⁡f^2^(*Au*)
*f*_4_(PK)⇒In⁡f^3^(*Au*)
*f*_5_(PK)⇒In⁡f^3^(*Au*)
*f*_1_(*COM*)∧*f*_1_(cipher)⇒In⁡f^4^(*A*).
